# Budget-aware local influence iterative algorithm for efficient influence maximization in social networks

**DOI:** 10.1016/j.heliyon.2024.e40031

**Published:** 2024-11-01

**Authors:** Lingfei Li, Yingxin Song, Wei Yang, Kun Yuan, Yaguang Li, Min Kong, Amir M. Fathollahi-Fard

**Affiliations:** a School of Management, Hangzhou Dianzi University, Hangzhou, 310018, China; b School of Economics and Management, Anhui Normal University, Wuhu, 241000, China; c School of Management, Hefei University of Technology, Hefei, 230009, China; d School of Health Service Management and the Hospital Management Institute, Anhui Medical University, Hefei, 230032, China; e Département d’Analytique, Opérations et Technologies de l’Information, Université de Québec à Montreal, 315, Sainte-Catherine Street East, H2X 3X2, Montreal, Canada; f New Era and Development in Civil Engineering Research Group, Scientific Research Center, Al-Ayen University, Nasiriyah, Thi-Qar, 64001, Iraq

**Keywords:** Budgeted influence maximization, Cost model, Proxy-based algorithm, Social networks

## Abstract

The budgeted influence maximization (BIM) problem aims to identify a set of seed nodes that adhere to predefined budget constraints within a specified network structure and cost model. However, it is difficult for the existing algorithms to achieve a balance between timeliness and effectiveness. To address this challenge, our study initially proposes a refined cost model through empirical scrutiny of Weibo's quote data. Subsequently, we introduce a proxy-based algorithm, i.e., the budget-aware local influence iterative (BLII) algorithm tailored for the BIM problem, aimed at expediently identifying seed nodes. The algorithm approximates the global influence by leveraging the user's one-hop influence and circumvents influence overlap among seed nodes via iterative influence updates. Comparative experiments involving eight algorithms across four real networks demonstrate the effectiveness, efficiency, and robustness of the BLII algorithm. In terms of influence spread, the proposed algorithm outperforms other proxy-based algorithms by 20%–255 % and reaches the state-of-the-art simulation-based approach by 96 %. In addition, the running time of the BLII algorithm is reasonable. Generally, the proposed cost model and BLII algorithm provide novel insights and potent tools for studying BIM problems.

## Highlights

• Empirical data informs a realistic cost model for individual user activation in social networks.
• A novel degree centrality-based algorithm swiftly selects seed nodes for the BIM problem, mitigating overlap.
• Experimental validation demonstrates our algorithm's efficiency and superior influence spread compared to alternatives.

## 1 Introduction

With the continuous advancement of information technology, social media platforms such as X, TikTok, Facebook, and Chinese Weibo have become an integral part of our daily lives. According to the Digital 2023 Global Overview Report, social media users now constitute more than 90 % of all Internet users and approximately 60 % of the global population. This exponential growth in user base has elevated social media to the forefront of information dissemination channels. Compared to traditional media, social media offers numerous advantages, including rapid propagation, minimal cost, extensive reach, targeted audience segmentation, measurable impact, and real-time communication capabilities [1,2]. Consequently, an increasing number of businesses are leveraging social networks for marketing purposes, a strategy commonly referred to as social marketing,^1^ with expenditure on social media advertising surpassing one-third of global digital advertising spending [3].

A significant hurdle in social marketing lies in identifying influential users, often referred to as “seed nodes,” who can effectively spread marketing content to a wide audience. This challenge aligns with the broader domain of influence maximization (IM), wherein the traditional approach involves fixing the number of seed nodes to a predefined value, typically denoted as “*k*" (e.g., 50). Consequently, research traditionally concentrates on identifying the top *k* users in terms of influence within the network, commonly referred to as “mega-influencers,” aiming to harness the extensive reach of their fan bases for effective propagation of marketing messages. However, this approach overlooks the variability in costs associated with persuading users to share information. Furthermore, activating mega-influencers often incur significantly higher costs than activating micro-influencers. Consequently, leveraging mega-influencers may exceed a company's marketing budget or yield less cost-effective outcomes [3]. Recent studies suggest that micro-influencers, renowned for their perceived authenticity and cost-efficiency, often provide more persuasive endorsements than mega-influencers [4]. In response to these challenges, budgeted influence maximization (BIM) has emerged as a solution. BIM introduces a scenario where each user in a social network incurs distinct activation costs. Within this framework, the BIM problem aims to identify the optimal set of seed nodes that not only adheres to budget constraints but also maximizes the propagation effect of the disseminated information.

This study aims to tackle two primary research questions. First, it endeavors to devise an efficient model for evaluating the costs associated with social media users. One motivation behind introducing the BIM problem is that users deemed influential based solely on their reach may not necessarily make the most cost-effective choice when budgetary constraints are factored into the problem. For instance, consider two users, A and B, where A's real cost is 100 with an expected influence spread of 1000, and B's real cost is 70 with an expected influence spread of 900. While A might appear to be the superior choice based on the influence spread alone, factoring in the budget reveals that B offers a more favorable unit-cost influence spread. Hence, the accurate modeling of user costs is paramount. A reliable cost model is an important prerequisite for solving BIM problems. Deviations between the real costs and model assumptions can lead to erroneous seed node selection, undermining the reliability of the resulting seed set and subsequent managerial decisions. However, although researchers have selected different cost models, there is no theoretical support for the selection of these models, and no empirical research can prove which type of cost model is the most realistic.

Second, this study aimed to devise an efficient algorithm for identifying the optimal set of seed nodes. Traditional optimization algorithms are ill-equipped to address this challenge owing to the NP-hard nature of the BIM problem. Greedy algorithms offer approximate solutions but struggle with scalability to large datasets. Heuristic algorithms, on the other hand, boast low time complexity and scalability but often sacrifice accuracy. Therefore, it is difficult for these algorithms to achieve a balance between efficiency and effect, making it difficult to apply the aforementioned theoretical research in concrete practice. Consequently, there is an urgent need to develop a robust and efficient model capable of accurately identifying the optimal seed nodes for BIM problems. Despite existing research in this domain, significant opportunities for further exploration and innovation remain unexplored.

In this study, we leveraged data extracted from Weibo to quantify the correlation between users' follower counts and their blog quotes by employing diverse functions to ascertain the optimal format for the activation cost function. Subsequently, we introduce a rapid algorithm called budget-aware local influence iterative (BLII), grounded in degree centrality, to address the BIM problem. Our experimental findings conducted across four authentic networks underscore the efficacy, efficiency, and resilience of the proposed algorithm.

The primary contributions of this paper can be delineated into three key aspects. First, by empirical research, we establish a robust cost model, informed by empirical data, to emulate the activation cost for individual users. This model provides a realistic framework for assessing the costs associated with the user activation of social networks. Second, we introduce a novel algorithm grounded in degree centrality that is tailored to address the BIM problem. This algorithm offers a swift and efficient approach to seed node selection, leveraging one-hop influence spread to approximate global influence while iteratively updating user influences to mitigate overlap among seed nodes. The algorithm can select seeds within a reasonable amount of time and obtain a better influence spread; hence, it can be extended to large-scale networks. Third, through a simulation study, we conduct a comprehensive comparative analysis by pitting the proposed algorithm against eight alternative approaches across four authentic network datasets. Our experimental findings corroborate the efficacy of the proposed algorithm, demonstrating its ability to swiftly generate seed sets while achieving competitive performance comparable to that of a greedy algorithm. Moreover, our algorithm outperformed the other seven algorithms in terms of influence spread, underscoring its efficacy and superiority in seed nodes selection.

The subsequent sections of this article are structured as follows. In Section 2, we delve into the existing body of related research. Section 3 describes the proposed cost model and offers insights into its construction and application. Section 4 describes the BLII algorithm designed to address the BIM problem and elucidates its underlying principles and operational mechanisms. Section 5 presents an extensive evaluation of the performance of the BLII model across four authentic network datasets, providing empirical evidence for its efficacy. Finally, Section 6 presents our findings and concludes.

## 2 Related work

The BIM problem is an extension of the IM problem, and its basic concepts and algorithms have the same origins as the IM problem. Therefore, in this section, we first provide an overview of the traditional IM problems, including information dissemination and IM algorithms. Subsequently, the BIM problem was reviewed.

### 2.1 Traditional IM problem

The IM problem was first described by Kampe et al. [5]. The formal definition of this problem is as follows: Given a social network G, an information propagation model, and a parameter k, we define σ(v), the influence spread of node v, as the expected total number of activated nodes during the propagation of a message sent from that node. The IM problem involves finding a set *S* with *k* nodes, such that the expectation of the total number of nodes that can be influenced by set *S* is maximized.

Two primary research avenues emerged from this foundational problem formulation: advancements in information propagation models and the development of IM algorithms.

#### 2.1.1 Information propagation models

The two classical information propagation models used for solving the IM problem are the independent cascade (IC) [5] and linear threshold (LT) models [6]. Both models assume that network G is a directed graph and that the direction of the edges is the direction of information propagation. Nodes in the network only have two states: active and inactive. The basic idea of the IC model is that each user has one chance to influence their inactive neighbors with a given probability after being activated, and such influences are independent of each other. The LT model posits that the effect of influence should be cumulative; that is, when a node is constantly influenced by a piece of information, its acceptance of that information gradually increases. Once a node's acceptance of information exceeds a certain threshold, it is activated.

Various studies have been conducted based on these fundamental models. A series of improved models have been developed that incorporate a broader array of factors, such as network structure [[7], [8], [9], [10]], information content [11,12], competitive relationships [[13], [14], [15], [16], [17]], and propagator characteristics [18]. Moreover, some researchers have attempted to jump out of the framework of the IC and LT models and have proposed novel information propagation models, such as the Gaussian propagation [19] and Hierarchical Attention Cascade Neural Network (CasHAN) models [20].

Despite ongoing advancements in information propagation modeling, the IC and LT models continue to be the predominant choices for addressing the IM problem.

#### 2.1.2 IM algorithms

Owing to its NP-hard nature, devising effective algorithms to tackle the IM problem has emerged as a central endeavor within this domain. Researchers have made a lot of attempts to solve this problem, and these algorithms can be classified into four groups.

**The first category is the *simulation-based approach*.** A representative algorithm in this category is the greedy algorithm proposed by Kampe et al. [5]. This algorithm employs Monte Carlo (MC) simulation techniques to simulate information propagation across individual nodes. It estimates the influence spread of individual nodes by calculating the average of the influence spread across multiple simulations (typically 10,000). Subsequently, the algorithm identifies the node with the greatest marginal influence during each iterative round and designates it as the seed node for that iteration. A number of algorithms have been proposed to reduce the number of MC simulations, such as the cost-effective lazy-forward ++ (CELF++) scheme [21]. The *simulation-based* algorithms have an approximation guarantee because the influence spread exhibits both monotonicity and submodularity within the IC and LT models. However, this type of algorithm is generally limited in reducing the effect of time complexity. However, scaling them into large-scale networks remains difficult.

**The second category is the *sketch-based approach*.** This approach mainly focuses on evaluating the influence spread by pre-computing several sketches based on the given graph and a specific diffusion model and then evaluating the influence spread based on these sketches. The most representative is the reverse influence sampling (RIS) algorithm proposed by Borgs [22]. The basic idea of this algorithm is that if a node frequently appears as an influencer, it is likely to be the most influential. Thus, the node that appeared most often in the reverse-reachable set was selected as the seed node. The algorithm achieves a constant-factor approximation for the IM problem. Subsequently, several improved algorithms were proposed, such as Influence Maximization via Martingales (IMM) [23], bottom-k sketch-based RIS (BKRIS) [24], and Attribute-based DIversity-sensitive Targeted Influence Maximization (ADITUM) [25]. These algorithms can reduce time complexity while preserving their approximation; however, they are still not realistically fast for large-scale networks.

**The third category is the *proxy-based approach*.** The core concept of this category is to adopt proxy models to approximate the influence spread of a given seed set S, thereby avoiding time-consuming MC simulations. Typical algorithms in this category are as follows. First, there are degree-based algorithms such as DegreeDiscountIC [26], Higher-order Augmented Random Walk (HoRW) [27], and Weighted Degree Decrease (WDD) [11]. Second, there are shell-based algorithms include *the k*-shell [28], *s*-shell [29], *k*_*s*_-shell [30], and shell-based ranking and filtering method (SRFM) [31]. Third, there are community-based algorithms in which the network is divided into several non-overlapping communities, and seed nodes are selected according to different proxy models in each community [8,[32], [33], [34], [35], [36], [37]]. These algorithms typically have low time complexity and can be scaled to large-scale networks; however, there are no approximation guarantees. Therefore, they should be compared with simulation-based approaches to demonstrate the effectiveness of the algorithm.

**The fourth category is the *intelligent optimization-based approach*.** The core idea of this category of algorithms is to introduce intelligent optimization algorithms, such as genetic algorithms [38,39] and simulated annealing approaches [40], to solve IM problems. With the wide application of deep learning, deep learning-based algorithms have been proposed in recent years [[41], [42], [43], [44], [45]].

Traditional IM problems have been researched for more than 20 years. Extensive research has been conducted on information propagation models and IM algorithms, and a series of meaningful results and conclusions have been obtained. However, because the traditional IM problem greatly simplifies real situations, the relevant conclusions cannot guide practice well. Many scholars have attempted to resolve this problem. For example, consider information content [46], propagation uncertainty [47], information overload [48], influence imbalance [49], and networks with unknown topology [50]. An important branch of this field is the BIM problem.

### 2.2 BIM problem

The BIM problem assumes that the budget is given and the activation cost of each user is different. Information propagation models for traditional IM problems can also be used for BIM problems as BIM is an extension of the IM problem. However, BIM introduces a new problem: how can a suitable cost model be constructed to fit the user's activation cost? Therefore, we provide an overview of the BIM problem in three parts: definition of BIM problems, BIM algorithms, and cost model.

#### 2.2.1 Definition of BIM problems

The BIM problem was first proposed by Nguyen et al. [51]. They defined the BIM problem as follows:

(Definition 1) Suppose each node u is associated with an arbitrary cost $C_{u}$. The goal of this problem is to select a seed set S such that the total cost of this set is less than a given budget B and the influence spread of the seed set S; that is, σ(S) is maximized.

However, the above definition of the BIM problem does not consider the fact that the number of seed nodes obtained by different seed node selection strategies is different for a given budget, and the number of seed nodes should not be counted in the influence spread. For example, strategy A activates 100 nodes, of which 2 nodes are seed nodes, and strategy B activates 101 nodes, 50 of which are seed nodes. If we use only the final influence spread, we should choose strategy B. However, if we exclude the seed nodes themselves, strategy A will obviously be better than strategy B. Based on the above considerations, Liu et al. [52] defined the BIM problem as follows:

(Definition 2) Consider a graph G, an information propagation model (say IC, LT, and so on), budget B, and the cost $C_{v}$ to activate node v. Let the expected influence of seed set S be σ(S)−k. The BIM problem requires a seed set S such that σ(S)−k is as large as possible, and the cost to activate the seed set is no more than the budget B. In this study, we selected Definition 2 as the definition of BIM.

#### 2.2.2 BIM algorithms

Because the two definitions of BIM problems differ only in terms of the influence spread, the algorithms proposed for these two types of problems can be interoperable with simple modifications. Here, we summarize the studies on these two types of problems. The aforementioned four types of strategies for solving the IM problem are also used to solve the BIM problem.

**Regarding simulation-based approaches**, according to definition 1, Nguyen et al. [51] proposed a greedy algorithm that can attain an approximation guarantee of (1–1/e) and then provide two heuristics to capture the bulk of the influence spread. According to definition 2, Liu et al. then proposed a simulation-based algorithm named GMUI, which obtains a solution that is provably $\left(\sigma \left(S_{k}\right)-k\right)\geq \left(1-1/e\right)\left(\sigma \left(OPT\right)-\omega \right)-\left(k-1\right)$. Notably, when the marginal influence per unit cost of a candidate node is below a given threshold, the selection of seed nodes should be stopped even though the firm still has a budget surplus. However, this type of algorithm is time-consuming.

**Regarding sketch-based approaches,** Bian et al. [53] applied a reverse-sampling technique to the BIM problem and proposed a framework with a greedy threshold and bound refinement. Two RR Sets are constructed using this framework. Then, seed node S is selected on the first RR Set and verifies whether S provides an approximation guarantee using the second RR Set. If *S* does not do so, the number of samples in these two RR sets doubles. This process is repeated until the approximation is satisfied. Using this strategy, Guney [54] applied a sample average approximation scheme to effectively solve the BIM problem. Similarly, they are not realistically fast for large-scale networks.

**Regarding proxy-based approaches,** Zhang et al. [55] proposed a local-global influence indicator-based constrained evolutionary algorithm that simultaneously considers the local influence, influence within communities, and influence among communities to calculate the node's final influence. Based on the proposed influence indicator, an adaptive initialization method was proposed to improve the seed diversity. Banerjee et al. [56] proposed a community-based solution approach for the BIM problem, called ComBIM, in which the seed set is obtained by dividing the budget into different communities and selecting the node that satisfies the budget and has the largest degree of centrality as the seed node within each community. Souza et al. [57] proposed a seeding approach based on the LT model to address this problem. The main idea of this approach is as follows: compared to direct seeding, central, expensive, or even cost-prohibitive nodes, targeting their cheapest neighbors can leverage their higher spreading potential at much lower costs. However, this algorithm cannot be applied to IC models.

**Among intelligent optimization-based approaches,** Yang et al. [58] proposed a multi-objective discrete particle swarm optimization algorithm to solve BIM problems. The last two approaches have lower time complexities, but the effect of the algorithms on different networks is difficult to guarantee.

Additionally, new application scenarios were extended based on the BIM. Considering that the retweeted offer is lower than the original offer (convincing a user to retweet a tweet is much less costly than getting the user to post the tweet), the holistic BIM (HBIM) has been proposed [59]. Assuming that each node is assigned a benefit value that can be obtained by influencing it, an earned benefit-maximization problem was proposed to determine a set of nodes to maximize the sum of benefit values under a given budget [60].

Although some researchers have researched BIM algorithms, it is difficult to achieve a balance between efficiency and effectiveness, which makes it difficult to apply the above theoretical research to specific practices. Therefore, the design of a fast and effective BIM algorithm is the focus of this study.

#### 2.2.3 Cost models

User activation costs often differ across online social networks. For example, finding a user with one million followers to disseminate a marketing message would certainly be more expensive than finding a user with only 100 followers to promote the message. Some studies assumed that user activation costs are randomized to measure cost variability [51,54,56,60]. However, this assumption does not match the actual scenario; therefore, studies further assume that the degree centrality of node u (usually the outdegree, denoted as $d_{u}$) is proportional to the activation cost (denoted as $C_{u}$); the greater the outdegree of a user, the greater the cost of activating that user. Guided by this simple principle, some scholars assume that the activation cost is a linear function of the outdegree [52,53,60]. In contrast, others believe that the activation cost is an exponential function of the outdegree [55,58,59]. Others believe that the activation cost is a power function of the outdegree [57]. The cooperation among the cost models is presented in Table 1. However, none of these hypotheses have been supported by empirical studies.

**Table 1:** Cooperation of cost models.

Type	Cost model	Literature
Random	Uniformly in [1.0, 3.0].	Nguyen et al. [51]
	Uniformly at random from the interval of [50,100]	Banerjee et al. [56]
	Randomly selected from [0,100]	Guney [54]
	Selected from the intervals [1,50]	Banerjee et al. [60]
Linear	The payment to influencers is assigned as $C_{u}=0.01∙d\left(v\right)$ and pay-rate to non-influencers is set as $C\left(v\right)=2∙d_{u}\text{.}$	Bian et al. [53]
	$C_{u}=\beta ∙d_{u}$	Liu et al. [52]
	$C_{u}=n\times d_{u}/2m$	Banerjee et al. [60]
Exponential	$C_{u}=m^{d_{u}/r}+d_{u}/r∙s$	Zhang et al. [55], Yang et al. [58]
	$C_{u}=\alpha ∙\left(1-e^{-\left(d_{u}+1\right)}\right)$	Shi et al. [59]
Power	$C_{u}={d_{u}}^{\alpha }$	Souza et al. [57]

Although researchers choose a lot of models as cost models in different studies, there is no theoretical literature that can support the selection of these models, and no empirical research can prove which kind of cost model is the most realistic. Reliable cost models are important prerequisites for solving BIM problems. When the cost model does not conform to the actual situation, the seed nodes excavated according to the model may be far from the optimal solution. Therefore, further research on the cost models is required.

In summary, although the IM problem has received extensive attention, the BIM problem requires further investigation. Improving the efficiency of the algorithm while ensuring its effectiveness remains under investigation. The construction of a reasonable cost model to ensure that the selected seed nodes are reasonable requires further research.

## 3 Constructing the proposed cost model

To investigate the correlation between users' activation costs and their out-degree, we focused on Weibo, a Chinese microblogging platform, and gathered cost data pertaining to Weibo users. Based on the above data, an empirical study was carried out. We employed linear, power, and exponential functions to model the relationship between a user's out-degree (i.e., the number of followers) and the associated costs. This analysis aimed to determine the optimal form of the cost model for our study.

### 3.1 Data description

The Microtask^2^ is an official Sina Weibo promotional platform. The platform has two primary roles: advertisers and we-media. Suppose a Weibo user possesses a substantial fan base, excels in content creation, and believes that they can effectively promote brands to drive sales. In that case, they may log in as we-Media on the Microtask platform and submit their quotations (cost). Upon approval of the quotation, Weibo users can commence receiving orders as Weibo masters. Similarly, suppose a Weibo user seeks promotion and publicity for their brand and wishes to collaborate with the Weibo master on the Microtask platform to enhance brand reputation. In that case, they can log in as advertisers. They can then review basic information and quotations from the Weibo master and select suitable individuals to promote their content.

**Fig. 1:**
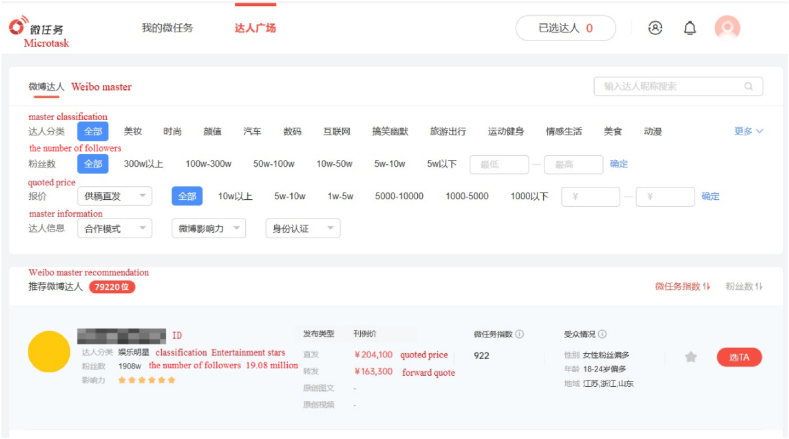
Screenshot of the Microtask webpage.

Fig. 1 shows a screenshot of the Microtask webpage. The webpage shows the basic information of each Weibo master, including ID, classification, number of followers (outdegree), and quoted price. The classification contained 37 categories, and the specific category information is shown in Table 2. In this study, we crawled the data of all 79,220 Weibo masters in Microtask by java crawler technology. Because Sina Weibo officials reviewed all quotes, we believe that they are valid. Therefore, we retained 79,220 records and did not screen them.

**Table 2:** Goodness of fit of three types of fitting functions.

ID	Category	$R^{2}$ linear	$R^{2}$ Exponential	$R^{2}$ Power	ID	Category	$R^{2}$ linear	$R^{2}$ Exponential	$R^{2}$ Power
1	Internet	0.1463	0.1725	**0.54**	20	Fashion and beauty	0.1507	0.1977	**0.5331**
2	Humanities and arts	0.0322	0.1229	**0.3837**	21	Horoscope and numerology	0.2483	0.2181	**0.461**
3	Sports	0.1084	0.1089	**0.3917**	22	Not available	0.3097	0.0783	**0.3348**
4	Health care	0.0712	0.1505	**0.4016**	23	Campus	0.0995	0.0751	**0.3742**
5	Public welfare	**0.8386**	0.271	0.4637	24	Mother and baby parenting	0.0349	0.1869	**0.4649**
6	Military	0.3968	0.2102	**0.5525**	25	Automobile	0.0352	0.0979	**0.3409**
7	Animation	0.0215	0.1084	**0.4244**	26	Law	0.0879	0.184	**0.4994**
8	Animals and pets	0.1548	0.1763	**0.4911**	27	Games	0.1138	0.1626	**0.4**
9	History	0.1083	0.1774	**0.4291**	28	Movies	0.1221	0.2177	**0.4178**
10	Entertainment stars	0.3547	0.1083	**0.4542**	29	TV shows	0.0407	0.2768	**0.4726**
11	Media	**0.9587**	0.3294	0.4671	30	Science and technology	0.054	0.1711	**0.5423**
12	Religion	0.0118	0.2294	**0.3542**	31	Technology and digital	0.0542	0.162	**0.4069**
13	Emotion	0.1024	0.1631	**0.4411**	32	Variety shows	0.0239	0.192	**0.4004**
14	Real estate and home	0.007	0.1108	**0.484**	33	Food	0.1006	0.1689	**0.4319**
15	Funny humor	0.3148	0.1584	**0.5246**	34	Reading and writers	0.0605	0.111	**0.5088**
16	Government	0.2269	0.3338	**0.5561**	35	Finance and economics	0.1162	0.1281	**0.3878**
17	Education	0.0842	0.1889	**0.5261**	36	Music performances	0.0252	0.1146	**0.4695**
18	Travel and tourism	0.047	0.1496	**0.4365**	37	Facial attractiveness	0.1165	0.1165	**0.3909**
19	Current affairs reviews	0.0273	0.0876	**0.3935**		**Average**	0.1569	0.1680	**0.4474**

### 3.2 Function fitting

We selected three types of fitting functions to fit the relationship between the user's out-degree and cost.

1 Linear function: Assume that the relationship between node u*’*s outdegree $d_{u}$ and cost $C_{u}$ is $C_{u}=a∙d_{u}+b$.
2 Exponential function: Assume that the relationship between $d_{u}$ and $C_{u}$ is $C_{u}=\beta^{d_{u}}$. Taking the logarithm of both sides of the equation yields $\log\left(C_{u}\right)=\log\left(\beta \right)∙d_{u}$. That is, the logarithm of the cost $\log\left(C_{u}\right)$ and the number of followers $d_{u}$ are linearly related.
3 Power function: Assume that the relationship between $d_{u}$ and $C_{u}$ is $C_{u}={k∙d_{u}}^{\alpha }$. Taking the logarithm of both sides of the equation yields $\log\left(C_{u}\right)=\alpha ∙\log\left(d_{u}\right)+\log\left(k\right)$. The logarithms of cost, $\log\left(C_{u}\right)$ and outdegree $\log\left(d_{u}\right)$ are linearly related.

After converting the three types of functions into linear forms, the least-squares method was used to fit the data for each category separately by MATLAB. The goodness-of-fit $R^{2}$ under the three regression functions are listed in Table 2.

The average goodness of fit for the 37 categories of sample data did not reach 0.9, regardless of whether they were fitted with a linear, exponential, or power function. This suggests that the cost to a user is affected not only by the number of users' followers. This is consistent with the intuitive understanding. The cost to a user may be related to other factors, such as the activity level of the user's followers, the number of retweets, comments, and likes on a user's previous posting, and the number of blogs posted by the user.

Nevertheless, the number of user followers remains one of the most important indicators of user cost. Given the question investigated in this study, what kind of positive relationship exists between cost and the outdegree of a user? We turned our focus back to the three fitting functions. Among them, the power function had the best fit, with an average goodness of fit of 0.4474, which was much larger than the average goodness of fit of the other functions. Therefore, assuming a power function relationship between the cost and outdegree is more reasonable; that is, $C_{u}={k∙d_{u}}^{\alpha }$.

**Fig. 2:**
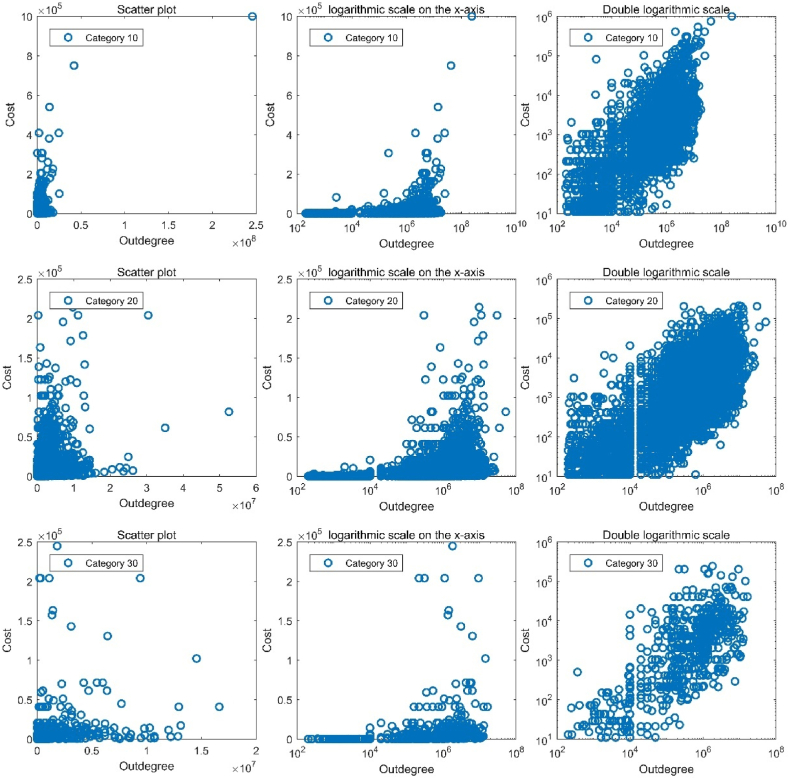
The relationship between outdegree and cost.

To intuitively demonstrate the rationality of choosing the power function as the cost model, we randomly selected the 10th, 20th, and 30th categories. We plotted the scatterplot of the relationship between the outdegree and cost under regular scales, a logarithmic scale on the x-axis, and a double logarithmic scale. The results are shown in Fig. 2. Thus, the relationship between the outdegree and cost is proportional and approximately linear on a double-logarithmic scale.

Table 3 demonstrates the fitting parameters for fitting the 37 categories of samples with the function $\log\left(C_{u}\right)=\alpha ∙\log\left(d_{u}\right)+\log\left(k\right)$, and sorted in descending order by α.

**Table 3:** The fitting parameters for fitting the 37 categories of samples.

Category	ID	α	log(k)	Category	ID	α	log(k)
Government	16	0.77	−2.7	History	9	0.57	−0.11
Media	11	0.73	−1.4	Internet	1	0.56	−0.35
Science and technology	30	0.69	−1.4	Humanities and arts	2	0.56	0.03
Law	26	0.67	−1.3	Horoscope and numerology	21	0.56	−0.65
Reading and writers	34	0.67	−1.2	Mother and baby parenting	24	0.56	−0.18
Music performances	36	0.67	−0.99	Food	33	0.55	0.014
Military	6	0.66	−1.7	Travel and tourism	18	0.54	0.14
Fashion and beauty	20	0.65	−1	Games	27	0.54	0.47
Funny humor	15	0.62	−1.2	Entertainment stars	10	0.53	0.065
Education	17	0.62	−0.81	Emotion	13	0.53	−0.14
Sports	3	0.6	−0.33	Current affairs reviews	19	0.53	0.23
Facial attractiveness	37	0.6	−0.045	Movies	28	0.51	0.36
Public welfare	5	0.59	−0.66	Religion	12	0.5	0.17
Real estate and home	14	0.59	−0.52	TV shows	29	0.5	0.38
Finance and economics	35	0.59	−0.3	Technology and digital	31	0.5	0.7
Animation	7	0.58	0.11	Variety shows	32	0.5	0.51
Animals and pets	8	0.58	−0.16	Campus	23	0.47	0.99
Automobile	25	0.58	0.03	Not available	22	0.45	0.76
Health care	4	0.57	−0.31				

Here, α is the power of the power function, which is estimated to be between 0.45 and 0.77. Categories with some of the largest α values of the fitted function are government, media, science and technology, and law. Content creators in the above categories usually have certain social credibility, represent authority and correctness, are mainstream, and have a professional background. According to the authority effect [61] and Hovland's persuasive and communication theory [62,63], the reliability of a spreader is based on two decisive factors: the audience's trust in the spreader and the credibility of the spreader. Thus, messages ascribed to high-credibility spreaders tend to be accepted to a greater extent than those ascribed to low-credibility spreaders. This makes these spreaders more cautious about their propagating behavior. Therefore, when the number of followers is the same, choosing the content creators in the above categories to publish information costs more than choosing other content creators. That is, it manifests itself as a larger α on the fitting function, or a larger slope under the double log linear fit. log(k) is the intersection of the fitting function with the y-axis. Analyzing the results of the 37 fitting functions reveals that α is approximately inversely related to log(k); the larger α, the smaller log(k). This is because when the outdegree of users is relatively small, the activation cost is similar for different categories of users. Thus, log(k) will be smaller when the slope α is larger. In summary, we assume that the relationship between the outdegree and cost is $C_{u}={k∙d_{u}}^{\alpha }$.

**Fig. 3:**
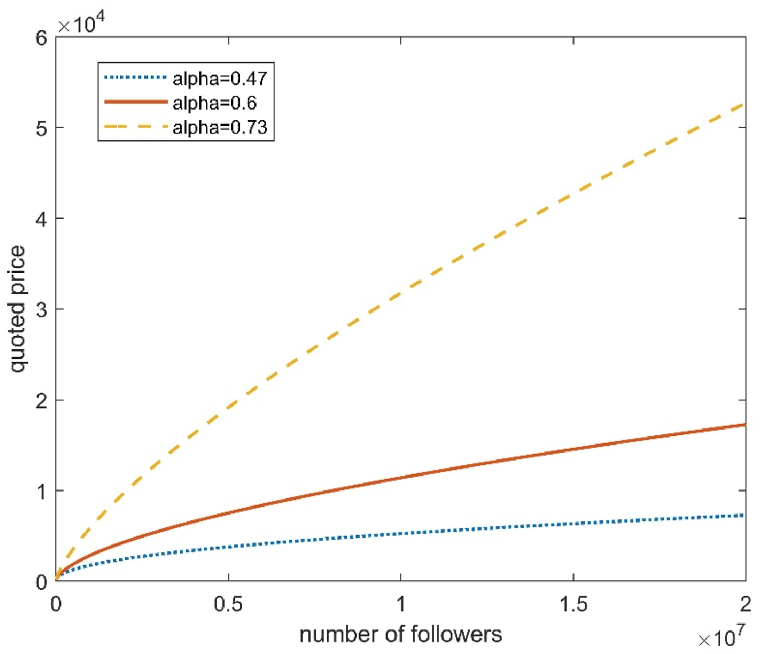
The fitted curves when α takes different values.

To illustrate the above conclusions more intuitively, three fitting curves with category ids 11 (Media), 3 (Sports), and 23 (Campus) are shown in Fig. 3. The power α of the three curves are 0.73, 0.6, and 0.47, respectively. The horizontal axis represents the number of followers, whose values range from 10,000 to 20 million, and the vertical axis represents the price quoted. As shown in Fig. 3, when the number of followers is small, say, 10,000, the corresponding quoted prices for the three curves are similar. However, as the followers' number increases, the larger the power α, the larger the corresponding quoted price. This result was consistent with the theoretical analysis.

When α is greater than 1, it means that the activation cost is greater than the number of followers and increases exponentially. In a realistic scenario, the probability of information being forwarded is far less than the number of times the information is read [11]. Therefore, α greater than 1 will lead to a high social marketing cost (which even exceeds the range of all enterprises) and a low expected net income, which is not cost-effective. For example, suppose a user has one million followers. If α = 1.5, the activating cost of the user reaches hundreds of millions ($10^{9}$); if α = 2, the activating cost of the user reaches hundreds of billions ($10^{12}$), which is obviously not practical.

## 4 Algorithms

Here, we introduce an extension to the BLII algorithm designed to expedite the process of identifying the seed set within the BIM problem. This extension builds on the foundation laid by Liu et al. [60], who initially proposed the BIM problem.

### 4.1 Budget-aware local influence iterative algorithm

The main concept of our BLII algorithm is as follows. The algorithm approximates the global influence of a node based on its one-hop influence, and the node with the largest marginal influence per unit cost is selected as the seed node for each iteration. This process is repeated until all budgets are exhausted or no budget-eligible candidate nodes exist. To avoid the overlap of influence spread among seed nodes, the one-hop influence of a node is iteratively adjusted in each iteration of the algorithm. If a node u is selected as a seed node, it is possible that its out-degree neighbor v will be activated by this node; thus, the marginal benefit that neighbor v can bring by selecting v as a seed node will be reduced accordingly. Simultaneously, the one-hop influence of all nodes that can spread information to u or v is reduced.

The steps of the BLII algorithm are as follows:

Let the information to be propagated be i, the maximum budget provided by the enterprise be B, and social network be G. In this regard, G is a directed graph, where the nodes are the users in the network, and the edges are the relationships between users. The edges are directed; if there exists an edge pointing from node u to node v, the information sent from user u can be propagated to user v. The weight of edge (u,v) is the probability p(u,v) that information i is propagated from u to v.

Each node u was assumed to have three corresponding metrics: $S_{u},F_{u}$, and ${\;C}_{u}$. $S_{u}$ denotes node u's contribution to itself after it is activated; that is, the probability value of node u remaining inactive. $F_{u}$ denotes the expected one-hop influence of node u. $C_{u}$ denotes the activation cost of node u. Without loss of generality, we assume that the relationship between $C_{u}$ and the out-degree $d_{u}$ of node u is as follows:

(1) $$C_{u}={\left(d_{u}\right)}^{m}$$

As given in Eq. (1), m is the power index. Based on the description in the previous section, m is assumed to be between 0.4 and 0.8. If node u is a content creator belonging to a category with greater social credibility, the value of m corresponding to that creator will be larger. For example, the value of m for users belonging to the government, media, law, and military categories is higher than that for users belonging to the emotion, game, and food categories. Souza et al. [57] used the cost model.

**Step 1. Initializing.** For each node u, we initialized $S_{u}$ as $S_{u}^{1}=1$. We then determine all the outdegree edges (u,v) of node u. For any outdegree neighbor v, we multiply its S-value $S_{v}$ by the weights of the edges (u,v), that is, p(u,v), and accumulate the above product to obtain the initial value of $F_{u}$ using the following formula:

(2) $$F_{u}^{1}=\sum_{v\in O\left(u\right)}p\left(u,v\right)\times S_{v}$$

As given in Eq. (2), O(u) denotes the set of out-degree neighbors of node u.

**Step 2. Seeding.** In each iteration, we first determine the node that satisfies the current residual budget as a candidate node and then determine the node that has the largest marginal influence per unit cost or node u which has the largest value of ${MI}_{u}^{t}=F_{u}^{t}/C_{u}$, as the seed node. Here, ${MI}_{u}^{t}$ is the marginal influence per unit cost of node u at time t. Then, we set the $S_{u}$ and $F_{u}$ of the current seed node u to 0 such that it is no longer involved in the subsequent seed node selection. Finally, we modify the remaining budget value for the next round as

(3) $$B^{t+1}=B^{t}-C_{u}$$

As given in Eq. (3), $B^{t}$ is the remaining budget at time t and $C_{u}$ is the activation cost of node u. If the value of $F_{u}^{t}$ is zero, let ${MI}_{u}^{t}$ = 0.

Because the computation of influence F is only related to the out-degree neighbors, the activated nodes themselves are not considered in the influence computation process.

**Step 3. Updating**S**.** We update the $S_{v}$ of an arbitrarily out-degree neighbor v of the current seed node. If node u is selected as a seed node at time t−1, its arbitrarily inactive out-degree neighbor v is likely to be activated at time t. Thus, the probability that v remains inactive at time t will decrease accordingly. $S_{v}$ of node v at time t is updated as follows:

(4) $$S_{v}^{t}=S_{v}^{t-1}-S_{v}^{t-1}\times p\left(u,v\right)$$

As given in Eq. (4), $S_{v}^{t}$ is the S value of node v at time t.

**Fig. 4:**
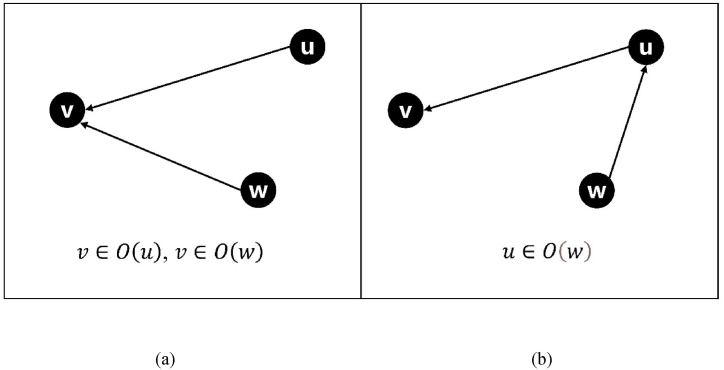
Relationship between nodes *u, v*, and *w*, (a): *u* and *w* are connected to *v* but not to each other; (b): *w* is connected to *u* where *u* is connected to *v*.

**Step 4. Updating F.** We updated the F values of all nodes. We assume that the seed node selected in this iteration is u. Because the F value of one node is related only to its out-degree neighbors, we only need to update the F value of the in-degree neighbors of the node whose S value has changed. That is, we should only update the F values of nodes u and $O{\left(u\right)}^{\prime }s$ in-degree neighbors (ignoring the seed nodes). That is, for any node w, if w is u’s indegree neighbor (u∈O(w)), or w is u’s outdegree neighbor v’s indegree neighbor (v∈O(u),v∈O(w)), we should update its $F_{w}$. This is illustrated in Fig. 4. As shown in Fig. 4(a), u and w are adjacent to v. If v is activated by u, then v should be ignored when calculating the expected influence of w. Therefore, the expected one-hop influence of w is reduced. In Fig. 4(b), w points to u, and u is activated, and the expected influence of w should decrease accordingly.

$F_{w}$ of node w at time t is updated as given in Eq. (5):

(5) $$F_{w}^{t}=\sum_{m\in O\left(w\right),m\neq u}S_{m}^{t}\times p\left(v,m\right)$$

**Step 5.** We repeat steps 2–4 until all budgets are exhausted or until no candidate nodes satisfy the budget conditions.

### 4.2 A concrete arithmetic example of BLII

**Fig. 5:**
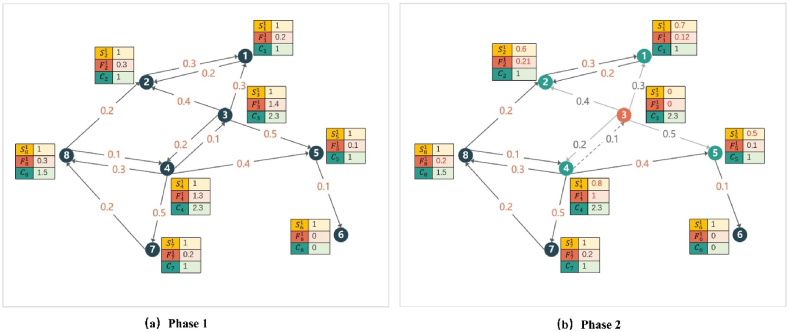
Numerical example for algorithm phases.

Below, we illustrate the algorithm through a concrete arithmetic example. Assuming that the network structure is as shown in Fig. 5 and budget B=4, the power index of the cost model is m=0.6, and the propagation probability for given information i is given in Fig. 5. Initially, the S value of each node is 1. In this regard, F values are as given in Eqs. (6), (7), (8), (9), (10), (11), (12), (13):

(6) $$F_{1}^{1}=p\left(1,2\right)\times S_{2}^{1}=0.2\times 1=0.2$$

(7) $${\;F}_{2}^{1}=p\left(2,1\right)\times S_{1}^{1}=0.3\times 1=0.3$$

(8) $$F_{3}^{1}=p\left(3,1\right)\times S_{1}^{1}+p\left(3,2\right)\times S_{2}^{1}+p\left(3,4\right)\times S_{4}^{1}+p\left(3,5\right)\times S_{5}^{1}=0.3+0.4+0.2+0.5=1.4$$

(9) $$F_{4}^{1}=p\left(4,3\right)\times S_{3}^{1}+p\left(4,5\right)\times S_{5}^{1}+p\left(4,7\right)\times S_{7}^{1}+p\left(4,8\right)\times S_{8}^{1}=0.1+0.4+0.5+0.3=1.3$$

(10) $$F_{5}^{1}=p\left(5,6\right)\times S_{6}^{1}=0.1\times 1=0.1$$

(11) $${\;F}_{6}^{1}=0$$

(12) $$F_{7}^{1}=p\left(7,8\right)\times S_{8}^{1}=0.2\times 1=0.2$$

(13) $$F_{8}^{1}=p\left(8,2\right)\times S_{2}^{1}+p\left(8,4\right)\times S_{4}^{1}=0.2\times 1+0.1\times 1=0.3$$

In addition, C values are defined as given in Eqs. (14), (15), (16), (17), (18), (19), (20), (21):

(14) $$C_{1}={\left(d_{1}\right)}^{0.6}=1^{0.6}=1$$

(15) $$C_{2}={\left(d_{2}\right)}^{0.6}=1^{0.6}=1$$

(16) $$C_{3}={\left(d_{3}\right)}^{0.6}=4^{0.6}=2.3$$

(17) $$C_{4}={\left(d_{4}\right)}^{0.6}=4^{0.6}=2.3$$

(18) $$C_{5}={\left(d_{5}\right)}^{0.6}=1^{0.6}=1$$

(19) $$C_{6}={\left(d_{6}\right)}^{0.6}=0$$

(20) $$C_{7}={\left(d_{7}\right)}^{0.6}=1^{0.6}=1$$

(21) $$C_{8}={\left(d_{8}\right)}^{0.6}=2^{0.6}=1.5$$

$S_{u}^{1},F_{u}^{1},\text{and}\;C_{u}$ for any node u in the first iteration are shown in Fig. 5(a).

All nodes in the first iteration satisfy the budget requirements, and the MI values per unit cost for each node are given in Eqs. (22), (23), (24), (25), (26), (27), (28), (29):

(22) $${MI}_{1}^{1}=\frac{0.2}{1}=0.2$$

(23) $${MI}_{2}^{1}=\frac{0.3}{1}=0.3$$

(24) $${MI}_{3}^{1}=\frac{1.4}{2.3}=0.61$$

(25) $${MI}_{4}^{1}=\frac{1.3}{2.3}=0.57$$

(26) $${MI}_{5}^{1}=\frac{0.1}{1}=0.1$$

(27) $${MI}_{6}^{1}=0$$

(28) $${MI}_{7}^{1}=\frac{0.2}{1}=0.2$$

(29) $${MI}_{8}^{1}=\frac{0.3}{1.5}=0.2$$

We select node 3 as the seed node in this iteration, set $S_{3}=0$ and $F_{3}=0$, and modify the remaining budget for the next iteration as given in Eq. (30):

(30) $$B^{2}=B^{1}-C_{3}=4-2.3=1.7$$

Next, we update the S value of the current seed node 3's out-degree neighbors 1, 2, 4, and 5 as given in Eqs. (31), (32), (33), (34):

(31) $$\text{Node}\;1:S_{1}^{2}=S_{1}^{1}-S_{1}^{1}\times p\left(3,1\right)=1-1*0.3=0.7$$

(32) $$\text{Node}\;2:S_{2}^{2}=S_{2}^{1}-S_{2}^{1}\times p\left(3,2\right)=1-1*0.4=0.6$$

(33) $$\text{Node}\;4:S_{4}^{2}=S_{4}^{1}-S_{4}^{1}\times p\left(3,4\right)=1-1*0.2=0.8$$

(34) $$\text{Node}\;5:S_{5}^{2}=S_{5}^{1}-S_{5}^{1}\times p\left(3,5\right)=1-1*0.5=0.5$$

S values of the other nodes remain unchanged.

The current seed nodes and their out-degree neighbors are 1, 2, 3, 4, and 5, and the in-degree neighbors of these nodes (except seed node 3) are 1, 2, 8, and 4. We update the F values of these nodes as given in Eqs. (35), (36), (37), (38):

(35) $$F_{1}^{2}=0.2\times 0.6=0.12$$

(36) $$F_{2}^{2}=0.3\times 0.7=0.21$$

(37) $$F_{8}^{2}=0.2\times 0.6+0.1*0.8=0.2$$

(38) $$F_{4}^{2}=0.3\times 1+0.5\times 1+0.4\times 0.5=1$$

F values of the other nodes remain unchanged.

The $S_{u}^{2},F_{u}^{2},\text{and}\;C_{u}$ for any node u in the second iteration are shown in Fig. 5(b).

In the second iteration, node 4 does not satisfy the budget requirement. The MI per unit cost for each node that satisfies the budget requirement is as given in Eqs. (39), (40), (41), (42), (43), (44):

(39) $${MI}_{1}^{2}=\frac{0.12}{1}=0.12$$

(40) $${MI}_{2}^{2}=\frac{0.21}{1}=0.21$$

(41) $${MI}_{5}^{2}=\frac{0.1}{1}=0.1$$

(42) $${MI}_{6}^{2}=0$$

(43) $${MI}_{7}^{2}=\frac{0.2}{1}=0.2$$

(44) $${MI}_{8}^{2}=\frac{0.2}{1.5}=0.13$$

We select node 2 as the current seed node, set $S_{2}=0\;and\;F_{2}=0$, and modify the remaining budget to the given in Eq. (45):

(45) $$B^{3}=B^{2}-C_{2}=1.7-1=0.7$$

No candidate node meets the remaining budget, and the algorithm terminates. The final set of seed nodes are nodes 3 and 2.

## 5 Experiments

To evaluate the effectiveness of the proposed BLII algorithm, we compared it with eight other algorithms using four real-world datasets through simulation experiments. All experiments were conducted using the IC model. We denoted the influence spread for each node as the total number of active nodes minus the number of seed nodes. To obtain the influence spread for each seed set, we ran the Monte Carlo simulation on each network 10,000 times and obtained the average influence spread over all the simulations as the final result. All simulation experiments were performed in C++ and were conducted on a machine with an Intel(R) Core(TM) i7-10510U CPU @ 2.30 GHz.

Without loss of generality, we set budget *B*=*300* and the power of the cost model *m* = 0.6. The probability of a given message propagating from user u to user v is the inverse of v’s indegree: $p\left(u,v\right)=1/{indegree}_{v}$.

### 5.1 Datasets

Next, we conduct experiments to test the proposed algorithm on four real-world social network datasets. All the datasets are publicly available.

**Coauthor:** This dataset is a topic-based co-authorship network. It is freely available in ArnetMiner [64]. Here, the nodes are authors, and the edges represent co-authorship.

**Facebook:** This is a social network crawled from Facebook and publicly available from the Stanford Large Network Dataset Collection.^3^ These statistics were compiled by combining the ego networks, including the ego nodes (along with an edge for each friend). The data include 4039 nodes and 88,234 edges.

**Digg:** This is a subset of the data scraped from Digg^4^ containing 8193 users and 56,440 explicit friendship relations among these users.

**Last.fm**: This is a subset of the Last.fm online music system.^5^ It contains 1892 users and 25,434 social relationships.

Table 4 presents the topological attributes of the datasets.

**Table 4:** Topological attributes of the datasets.

Dataset	Nodes	Edges	Average degree	Max degree	Average path length	Density
Coauthor	4229	14,961	7.075	97	6.118	0.002
Facebook	4039	88,234	43.691	1045	3.693	0.011
Digg	8193	56,440	12.775	645	3.371	0.002
Last.fm	1892	25,434	13.443	119	3.519	0.007

### 5.2 Comparison of algorithms

We compared the influence spread of our method with those of eight other algorithms. The details of the comparison algorithms are as follows.

➢ **GMUI** [52]**:** This is a greedy algorithm for BIM with the CELF optimization proposed by Liu et al. and can yield a solution that is provably $\left(\sigma \left(S_{k}\right)-k\right)\geq \left(1-1/e\right)\left(\sigma \left(OPT\right)-\omega \right)-\left(k-1\right)$. For each candidate seed set, 10,000 simulations were performed to obtain an accurate estimate of the influence spread.
➢ **ComBIM** [56]**:** This is a community-based BIM algorithm. The total budget is allocated to each non-overlapping community. High-degree nodes satisfying budget constraints are then selected within each community until the budget is exhausted.
➢ **BLII_S:** This is a variant of our BLII algorithm that iteratively selects the nodes with the highest (F + S)/C value as seed nodes until the budget is exhausted. In this algorithm, seed nodes are counted in the influence spread.
➢ **Degree**: This is a simple heuristic that selects nodes with the largest out-degrees as seed nodes until the budget is exhausted.
➢ ***k-*shell** [28]: This is a simple heuristic algorithm that selects nodes with the largest *k-*shell values as seed nodes until the budget is exhausted.
➢ ***k***_***s***_**-shell** [30]: This is a heuristic algorithm based on *k*-shell. This algorithm splits the core-like group by removing links with a diffusion importance of less than two. We identified the influential nodes in the remaining graph using a *k*-shell until the budget was exhausted.
➢ ***s*-shell** [29]**:** This is an extension of the *k*-shell method that uses a weighted degree to measure the weighted coreness of a node. We selected the nodes with the largest *s*-shells as seed nodes until the budget was exhausted.
➢ **Random**: This simple strategy randomly selects the nodes in a graph until the budget is exhausted.

### 5.3 Experimental results

Next, we evaluated the performance of the proposed and comparison algorithms on the described datasets in terms of the influence spread, sensitivity analysis, and run time.

#### 5.3.1 Influence spread

**Fig. 6:**
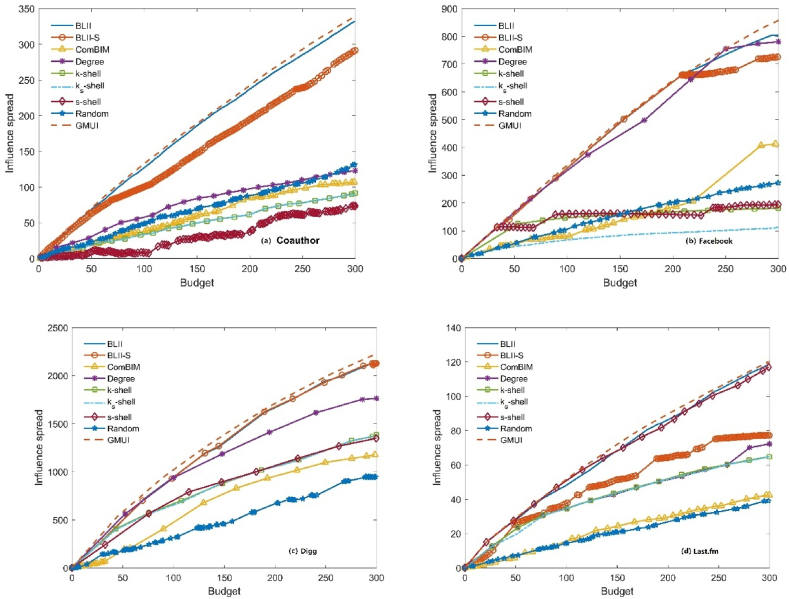
Influence spread of different algorithms.

The results of the influence spread are shown in Fig. 6. The horizontal axis represents the budget for activating the seed nodes, and the vertical axis represents the influence spread of the seed nodes.

The proposed BLII algorithm outperformed the other proxy-based algorithms and yielded a performance similar to that of the simulation-based GMUI algorithm in terms of the influence spread on all examined networks. Among the other algorithms, degree performed well on the Facebook network, BLII-S performed similarly to BLII on the Digg network, and s-shell performed well on the Last.fm network. However, these algorithms exhibit poor performance on other networks, indicating that they are not robust to different networks. Finally, ComBIM, *k*-shell, *k*_*s*_-shell, and random performed poorly on all the four examined networks.

Notably, in all four networks, BLII outperformed BLII-S. This suggests that if a strategy counts the seed nodes in the influence spread to find the seed nodes, the algorithm tends to find users with low influence but low cost as seed nodes. However, the enterprise activates these nodes themselves, and their activation does not generate revenue for the enterprise; thus, it is not a superior strategy under the BIM problem in this study. The similar influence spread of BLII-S and BLII in the Digg network (Fig. 6(c)) may be because of a small number of nodes in the Digg network that have greater influence. Thus, their influence spread per unit cost is higher than that of other nodes, regardless of whether the seed nodes are included in the influence spread. Thus, both algorithms essentially select the same seed nodes.

The number of seed nodes in the Coauthor network (Fig. 6(a)) exceeded those in the other networks. This may be because the Coauthor network had the smallest density, smallest maximum degree (97), and the longest average path length among the four networks. These topological features suggest that the connections between nodes in the Coauthor are relatively sparse and that there are no centrality nodes with an absolutely high influence. In such networks, the seed nodes are usually a large number of micro-influencers with low activation costs.

On Facebook (Fig. 6(b)), the number of seed nodes was relatively small. In addition to BLII and GMUI, the degree algorithm performed well. This may be because Facebook is an ego-oriented network. The number of high-centrality nodes in the network has a much greater influence than other nodes (the maximum degree is 1045, which covers approximately 25 % of the users in the network). The activation of these nodes is costly; thus, algorithms such as GMUI, BLII, BLII-S, and degree are chosen to activate nodes with high centrality as seed nodes.

In the Last.fm network (Fig. 6(d)), s-shell performed well; however, it did not perform well on other networks. This is because the effectiveness of the s-shell algorithm is highly dependent on the network structure. High s-shell nodes with the same s-shell values are typically closely connected. Therefore, the seed nodes selected by the s-shell algorithm may suffer from an influence overlapping problem, rendering the algorithm ineffective in most networks and achieving satisfactory results in only a few.

Subsequently, we synthesized the performance of the BLII algorithm for the four networks and compared the average values of the influence spread of each algorithm on the four networks. As a proxy-based algorithm, the influence spread of the BLII algorithm reaches that of the GMUI [52], which is a state-of-the-art simulation-based approach, by 96 %. It outperforms ComBIM [56], an advanced proxy-based algorithm for comparison, by 141 % and outperforms other comparison algorithms by 20%–255 %. This indicates that the BLII algorithm proposed in this study has a positive effect on the influence spread.

#### 5.3.2 Sensitivity analysis

We verified that the proposed algorithm was effective and reliable for different types of networks by selecting four types of networks and repeating the Monte Carlo simulation 10,000 times. The seed set in a BIM problem may differ substantially when the budget changes. For example, when the budget is low, users with a high outdegree in the network cannot be activated. Thus, all strategies find micro-influencers with a low outdegree as seed nodes. Theoretically, when the budget value is large, any node in the network can be activated, and all strategies may yield similar results. To further avoid research bias and to verify the robustness of the proposed algorithm under different budget values, we assume that the budget is 100 and 500 and compare the proposed BLII algorithm with eight algorithms on four networks again; the results are shown in Table 5.

**Table 5:** Comparison of influence spread when B = 100 and 500.

	Budget	BLII	BLII-S	Degree	Random	GMUI	ks-shell	k-shell	s-shell	ComBIM
Coauthor	100	**126.28**	100.119	57.9741	45.3742	**134.106**	33.59	33.81	8.1963	41.5815
	500	**504.697**	377.243	186.361	217.609	**514.411**	140	153.936	136.23	215.131
Facebook	100	**301.312**	292.208	324.847	111.563	**331.769**	64.88	148.013	162.448	93.221
	500	**977.87**	785.224	910.744	398.064	**1008.81**	135.78	194.544	503.73	789.361
Last.fm	100	**47.75**	37.3241	34.13	14.95	**54.2607**	34.65	34.91	50.0567	15.9859
	500	**172.139**	95.097	110.401	79.605	**170.744**	89.17	97.348	164.477	66.308
Digg	100	**937.02**	934.725	898.354	570.291	**953.652**	664.09	653.273	645.135	555.256
	500	**2916.69**	2912.87	2427.66	1043.34	**2953.1**	1719.03	1721.96	1757.12	1767.81

The proposed BLII algorithm was still the best algorithm, except for the GMUI when B = 100 and B = 500. Thus, it was the best among the eight proxy-based algorithms.

The above experiments show that the proposed BLII algorithm achieves better results in terms of influencing the spread under different networks and budgets.

#### 5.3.3 Running time

**Fig. 7:**
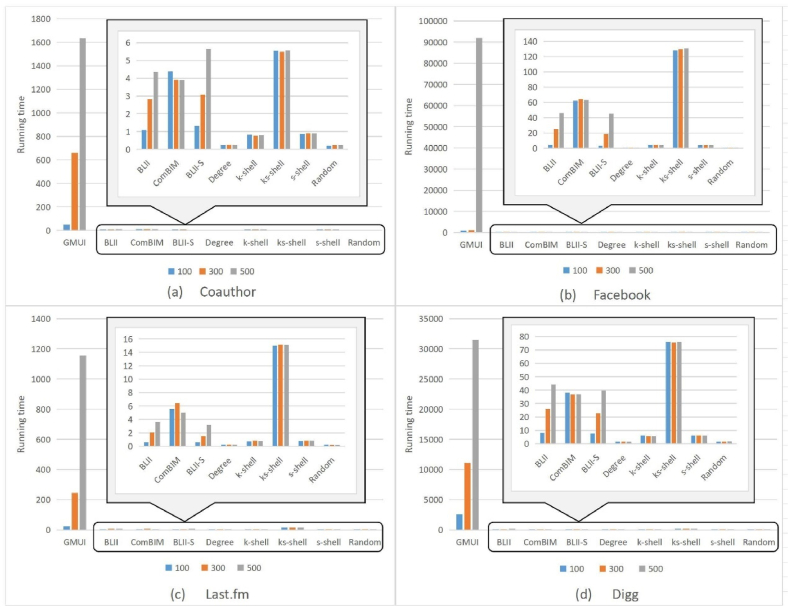
The running times of the nine algorithms for B=100,300,and500.

The running times of the nine algorithms for B=100,300,and500 are shown in Fig. 7.

The vertical axis indicates running time (*s*). Because the GMUI is a simulation-based algorithm, its time complexity is much higher than those of the other eight proxy-based algorithms. Among the eight proxy-based algorithms, degree and random had the shortest runtimes, followed by k-shell and s-shell algorithms. BLII outperformed BLII-S and ComBIM, whereas *k*_*s*_-shell had the longest runtime. Thus, the proposed BLII algorithm can obtain the seed node set in a shorter time and is time efficient.

In summary, the BLII algorithm can locate a set of seed nodes within a given budget in a short time. Moreover, the influence spread of this seed set can yield performance similar to that of the GMUI algorithm under different networks and budgets and outperforms other comparative algorithms. Thus, the proposed WDD algorithm was effective, efficient, and robust.

## 6 Conclusions and future works

In conclusion, this study addressed the challenge of identifying optimal seed nodes within BIM problem. Through an empirical analysis of quotation data from Weibo, we established that the relationship between a node's cost and its outdegree approximates a power function. Leveraging this insight, we develop a robust cost model and propose the BLII algorithm to expedite seed node identification.

The BLII algorithm estimates a user's global influence based on their one-hop influence. It synchronously updates the node influences at each iteration phase to mitigate the overlapping influence spread among the selected seed nodes. Through comparative experimentation on four real networks, our proposed BLII algorithm outperformed eight alternative algorithms, demonstrating superior efficiency in finding seed nodes that meet budgetary constraints. Notably, the influence spread of the BLII algorithm achieved GMUI [52] by 96 % and outperformed ComBIM [56] by 141 %.

Theoretically, this study proposes a more reasonable cost model through empirical analysis and proves that it is reasonable in theory. It can provide model support for subsequent studies on the BIM problem and avoid the distortion of seeds caused by the blind selection of the cost model. Moreover, the BLII algorithm proposed in this paper is both timely and effective, which is a beneficial attempt to solve BIM problems using a proxy-based approach. Third, our findings underscore the potential of leveraging micro-influencers, identified through the BLII algorithm, as cost-effective alternatives to traditional mega-influencers in social marketing campaigns. This can support further research on the internal relationship between seed nodes and network structure.

Practically, the low timeliness and unguaranteed results of the existing BIM algorithms make them difficult to apply in real marketing situations. The proposed algorithm can quickly recommend seeds to enterprises according to a reasonable cost model and a given marketing budget. The relevant conclusions are practical and feasible. Specifically, when an enterprise wants to carry out social marketing, given the budget and different network structure data as input, BLII can quickly and effectively determine which users should be selected as seed nodes for the above-mentioned budget under different networks and estimate the propagation effect of seeds on different networks to guide enterprises to make social marketing decisions.

This study still has some limitations. It empirically analyzed the cost model using Weibo data. Unfortunately, due to the lack of access to quotation data from other online social networks, we were unable to verify the universality of this cost model through a broader range of empirical studies. Additionally, the structure of real social networks is dynamic [65,66], and the cost to users is constantly changing. However, this study does not consider the dynamic evolution of the network. Future research could explore extensions of the BLII algorithm to accommodate dynamic network structures and evolving cost landscapes, enhancing its applicability in real-world scenarios. Investigating the integration of machine learning techniques [67], swarm algorithms [68], and real-time analytics [69] to refine cost models and improve algorithmic performance represents a promising direction for further advancement in this domain. Finally, our experiments show that the same cost can activate different numbers of users and lead to varying influence spreads across different networks. It would also be interesting to further study the relationship between network structure, seed characteristics, and influence spread.

## CRediT authorship contribution statement

**Lingfei Li:** Software, Methodology, Investigation, Formal analysis, Data curation, Conceptualization. **Yingxin Song:** Visualization, Validation, Supervision, Resources, Conceptualization. **Wei Yang:** Writing – original draft, Validation, Software, Methodology, Investigation, Formal analysis. **Kun Yuan:** Writing – original draft, Validation, Resources, Methodology, Investigation, Conceptualization. **Yaguang Li:** Writing – original draft, Visualization, Resources, Methodology, Investigation. **Min Kong:** Writing – review & editing, Writing – original draft, Resources, Project administration, Methodology, Conceptualization. **Amir M. Fathollahi-Fard:** Writing – review & editing, Writing – original draft, Visualization, Validation, Resources, Methodology, Conceptualization.

## Data availability statement

The data that has been used is confidential.

## Declaration of competing interest

The authors declare the following financial interests/personal relationships which may be considered as potential competing interests: The corresponding author, Prof. Amir M. Fathollahi-Fard, is an Associate Editor in Information Science for Heliyon and was not involved in the editorial review or the decision to publish this article. If there are other authors, they declare that they have no known competing financial interests or personal relationships that could have appeared to influence the work reported in this paper.

## Footnotes

1 https://datareportal.com/reports/digital-2023-global-overview-report.

2 https://weirenwu.weibo.com/wrw/?c=index.home#_loginLayer_1708484821706.

3 https://snap.stanford.edu/data/#socnets.

4 https://www.aminer.cn/heterinf.

5 https://grouplens.org/datasets/hetrec-2011/.
